# Malignant bowel obstruction related to advanced peritoneal metastases: survival outcomes of surgical treatment

**DOI:** 10.1515/pp-2025-0024

**Published:** 2026-04-07

**Authors:** Susanna Scarsi, Hugo Teixeira Farinha, Amaniel Kefleyesus, Martin Hübner, Daniel Clerc

**Affiliations:** Department of Visceral Surgery, 30635 Lausanne University Hospital (CHUV), University of Lausanne (UNIL), Lausanne, Switzerland; Department of Surgery, Centre Hospitalier du Valais Romand (CHVR), Hôpital de Sion, Sion, Switzerland

**Keywords:** peritoneal metastases, peritoneal surface malignancies, malignant bowel obstruction, palliative surgery

## Abstract

**Objectives:**

Malignant bowel obstruction (MBO) caused by advanced peritoneal metastases (PM) carries a poor prognosis. Surgical intervention may be the only therapeutic option in selected cases, but operative risks must be carefully balanced against potential benefits. This study aimed to evaluate the outcomes of patients undergoing surgery for MBO secondary to PM.

**Methods:**

Single-centre retrospective analysis of consecutive patients operated for MBO of various origins between 2016 and 2021. The primary outcome was overall survival (OS). Secondary outcomes included postoperative morbidity, resumption of systemic chemotherapy, and incidence of re-obstruction.

**Results:**

A total of 27 patients (median age 64 years, 67 % female) were included. Median peritoneal cancer index (PCI) was 32, and ascites was present in 16 patients (59 %). Surgical resolution of obstruction was achieved in 24 patients (88 %) via bowel resection (n=10), internal bypass (n=7), stoma formation (n=5), or adhesiolysis (n=2). Severe morbidity occurred in 26 %, with no postoperative mortality. Five patients (19 %) required reoperation, and three (11 %) developed enterocutaneous fistulae. Median OS was 4.0 months (IQR 9.4). Survival rates at 3, 6, and 12 months were 56 , 37, and 26 %, respectively. Postoperative systemic chemotherapy was resumed in 19 patients (70 %), significantly more often among those surviving >6 months (p=0.02). Re-obstruction occurred in 14 patients (52 %).

**Conclusions:**

Surgery is a feasible and valid therapeutic option for selected patients with MBO due to advanced PM. Despite considerable morbidity, most patients are able to resume systemic chemotherapy, which may contribute to improved survival outcomes.

## Introduction

Malignant bowel obstruction (MBO) refers to a bowel obstruction caused by a neoplastic process, most commonly resulting from extrinsic compression by peritoneal metastases (PM). PM can arise from various primary tumors and often progresses within the abdominal cavity, eventually leading to obstruction in an estimated 28–51 % of cases [1], 2].

MBO secondary to PM presents a substantial therapeutic challenge and is associated with a poor prognosis. Most patients are not candidates for complete cytoreductive surgery (CRS) due to the extent of disease and the emergency nature of presentation. Additionally, systemic chemotherapy is typically contraindicated in the presence of bowel obstruction because of the elevated risk of gastrointestinal perforation. In the absence of treatment, median overall survival (OS) is limited to approximately 1–2 months [3].

The first-line management of MBO is generally conservative and includes bowel rest, nasogastric decompression, antisecretory agents, and corticosteroids [4], 5]. When symptoms persist despite optimal medical therapy, surgical intervention may be the only remaining therapeutic option. However, surgery in this context is technically demanding and carries a high risk of perioperative morbidity. Patients frequently present in poor general condition due to advanced oncologic disease, cachexia, and malnutrition. This raises an ethical dilemma for the clinical team: surgical intervention may lead to significant morbidity and may result in the patient spending their remaining lifespan in the hospital setting [3], 6], 7].

To date, the role of surgery in managing MBO due to PM remains poorly defined. We hypothesized that surgical exploration, with the intent to relieve obstruction, may offer meaningful clinical benefit in carefully selected patients. The aim of this study was to evaluate the outcomes of patients undergoing surgery for MBO secondary to PM and to assess whether surgery represents a viable therapeutic option in this population.

## Methods

This single-centre retrospective cohort study was conducted at the Department of Visceral Surgery, Lausanne University Hospital (CHUV), Switzerland. All consecutive patients undergoing surgery for MBO secondary to PM of various origins between January 2016 and July 2021 were included. Patients whose obstruction was due to primary tumor involvement were excluded. Surgical candidacy was determined on a case-by-case basis, considering performance status (ECOG 0–2), limited ascites (<500 mL), and an estimated life expectancy of more than 3 months. Demographic, intraoperative, and postoperative data were extracted from a prospectively maintained institutional database. Pre-treatment history, including prior intraperitoneal chemotherapy (PIPAC or HIPEC), was collected. Follow-up was completed through outpatient clinic visits and correspondence with referring oncologists or general practitioners and concluded in March 2022.

The primary outcome was overall survival (OS), defined as the time from surgery to death. Secondary outcomes included postoperative morbidity (graded using the Clavien–Dindo classification), resumption of systemic chemotherapy, and incidence of re-obstruction (defined as recurrent MBO requiring inpatient management). Patients were stratified into two groups based on survival: OS <6 months and OS ≥6 months.

Surgical candidacy was determined on a case-by-case basis within a multidisciplinary board, considering performance status (ECOG 0–2), limited ascites (<500 mL), and estimated life expectancy >3 months. Patients not fulfilling these criteria were managed conservatively or received best supportive care, including palliative PEG placement when indicated. This study was approved by the local ethics committee (CER-VD, #2019-00747).

### Statistical analysis

Descriptive statistics are presented as frequencies (%) for categorical variables and medians with interquartile ranges (IQR) for continuous variables. Comparisons between categorical variables were performed using Chi-square or Fisher’s exact tests. Continuous variables were compared using Student’s *t*-test for normally distributed data and the Mann–Whitney U test for non-parametric comparisons. All statistical analyses were two-sided, and a p-value <0.05 was considered statistically significant.

## Results

### Patient characteristics

A total of 27 patients were included in this study. Baseline characteristics were comparable between survival groups (Table 1). Twenty-three patients (85 %) had received prior systemic chemotherapy. Among them, 83 % (19/23) had undergone at least two lines of treatment. Prior intraperitoneal chemotherapy (PIPAC or HIPEC) was recorded in a minority of patients, as detailed in Table 1. Nutritional status was assessed by prealbumin levels, with a median value of 13.5 mg/dL (normal: 15–35 mg/dL), suggesting malnutrition across the cohort, similar between groups (p=0.46).

**Table 1:** Patients’ demographics.

	Overall (n=27)	OS >6 months (n=10)	OS <6 months (n=17)	p-Value
Age	64 (11)	62 (11)	64 (12.5)	0.34
Gender female	18 (67 %)	6 (60 %)	12 (71 %)	0.57
BMI, kg/m^2^	21.1 (5.1)	21.4 (3.5)	21 (6.2)	0.38
ASA, III-IV	18 (67 %)	6 (60 %)	13 (71 %)	0.37
PM origin				
– *Colorectal*	8 (30 %)	3 (30 %)	5 (29 %)	0.97
– *Appendix*	1 (4 %)	–	1 (6 %)	–
– *Ovarian*	10 (37 %)	4 (40 %)	6 (35 %)	0.81
– *Pseudomyxoma peritonei*	1 (4 %)	1 (10 %)	0 (0 %)	–
– *Other*	7 (26 %)	2 (20 %)	5 (29 %)	0.59
Previous surgery	20 (74 %)	7 (70 %)	13 (76 %)	0.71
Previous CRS-HIPEC	1 (4 %)	–	1 (6 %)	–
Previous PIPAC	5 (19 %)	2 (20 %)	3 (18 %)	0.89
Previous systemic chemotherapy	23 (85 %)	8 (80 %)	15 (88 %)	0.56
Number of previous chemotherapy lines				
−*1*	4 (15 %)	1 (10 %)	3 (18 %)	0.59
−*2*	10 (37 %)	4 (40 %)	6 (35 %)	0.81
– ≥*3*	9 (33 %)	3 (30 %)	6 (35 %)	0.78

Data expressed as n (%) or median (IQR). OS, overall survival; BMI, body mass index; ASA, american society of anesthesiologists; PM, peritoneal metastases; CRS, cytoreductive surgery; HIPEC, hyperthermic intraperitoneal chemotherapy; PIPAC, pressurized intraperitoneal aerosol chemotherapy.

### Surgical findings

Details of surgical procedures are summarized in Table 2. The small bowel was the most common site of obstruction, with similar distributions across survival groups. The median Peritoneal Cancer Index (PCI) was 32 (IQR=15.5), with no significant difference between groups (30 vs. 34, p=0.99). Ascites was present in 59 % (n=16) of patients.

**Table 2:** Surgical details.

	Overall (n=27)	OS >6 months (n=10)	OS <6 months (n=17)	p-Value
Obstruction type				
– *Small bowel*	13 (48 %)	5 (50 %)	8 (47 %)	0.88
– *Large bowel*	7 (26 %)	2 (20 %)	5 (29 %)	0.59
– *Both*	7 (26 %)	3 (30 %)	4 (24 %)	0.71
PCI	32 (15.5)	30 (18)	34 (16.5)	0.99
Ascites	16 (59 %)	4 (40 %)	12 (71 %)	0.12
Surgical duration, min	161 (131)	192 (112)	140 (162)	0.10
Surgical resolution of obstruction	24 (88 %)	10 (100 %)	14 (82 %)	–
– *Resection*	10 (37 %)	5 (50 %)	5 (29 %)	0.28
– *Internal bypass*	7 (26 %)	3 (30 %)	4 (24 %)	0.71
– *Stoma formation*	5 (19 %)	1 (10 %)	4 (24 %)	0.38
– *Adhesiolysis*	2 (7 %)	1 (10 %)	1 (6 %)	0.69
– *Indwelling peritoneal drainage*	3 (11 %)	0 (0 %)	3 (18 %)	–

Data expressed as n (%) or median (IQR). PCI, peritoneal carcinomatosis index.

Surgical resolution of obstruction was achieved in 88 % (n=24) of patients through various procedures: bowel resection (n=10), internal bypass (n=7), stoma formation (n=5), and adhesiolysis (n=2). In three patients (11 %), resolution was not possible, and peritoneal drainage catheters were placed for symptomatic relief. Intraoperative complications occurred in 37 % of cases, primarily due to iatrogenic bowel perforations (n=9).

### Postoperative outcomes

Postoperative outcomes are detailed in Table 3. Complications occurred in nine patients (33 %), including seven with major morbidity (Clavien–Dindo ≥ IIIa, 26 %). No postoperative deaths were recorded. Five patients (19 %) required reoperation, and three (11 %) developed enterocutaneous fistulae.

**Table 3:** Postoperative outcomes.

	Overall (n=27)	OS >6 months (n=10)	OS <6 months (n=17)	p-Value
LOS, days	12 (8)	13 (9)	12 (8)	0.42
Morbidity, overall	9 (33 %)	4 (40 %)	5 (29 %)	0.57
– *Severe morbidity (Clavien* ≥ *IIIa)*	7 (26 %)	3 (30 %)	5 (29 %)	0.97
– *Enterocutaneus fistula*	3 (11 %)	1 (10 %)	2 (12 %)	0.89
Return to theatre	5 (19 %)	2 (20 %)	3 (18 %)	0.88
Post-operative systemic chemotherapy	19 (70 %)	9 (90 %)	10 (59 %)	0.09
Recurrence of obstruction	14 (52 %)	4 (40 %)	9 (53 %)	0.52

Data expressed as n (%) and median (IQR). LOS, length of hospital stay.

The median length of hospital stay was 12 days (IQR=8), with no significant difference between groups. The majority of patients were discharged home (n=16, 59 %). Eight patients (30 %) were discharged to palliative care facilities; of these, seven survived less than six months.

Re-obstruction occurred in 52 % (n=14) of patients, with no significant difference in incidence between survival groups.

### Survival outcomes

**Figure 1:**
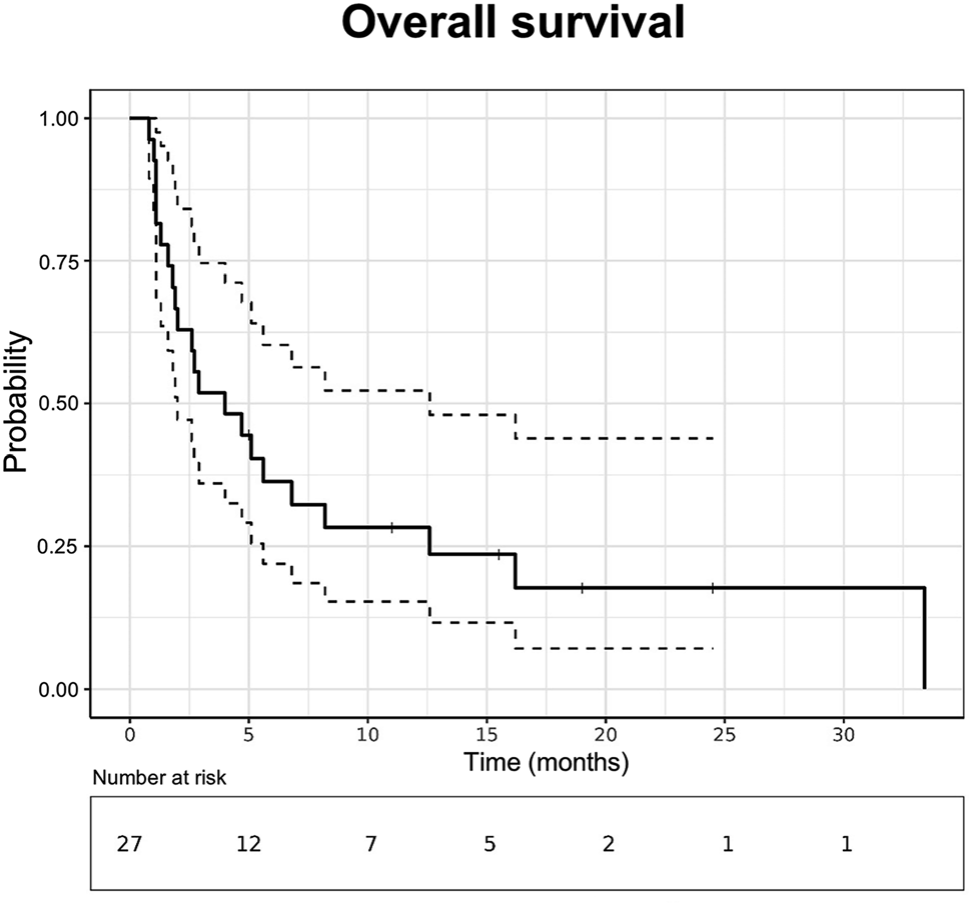
Cohort’s overall survival. Cohort’s overall survival (OS) estimated by the Kaplan–Meier method (solid line) with 95 % confidence intervals (dashed lines). Median OS: 4 months (IQR=9.4).

Median OS was 4.0 months (IQR=9.4) (Figure 1). Survival rates at 3, 6, and 12 months were 56 , 37, and 26 %, respectively. Patients surviving ≥6 months had a median OS of 14 months (IQR=8.5), compared to 2 months (IQR=2.3) for those in the <6 months group. Within the longer survival group, 70 % (n=7) survived ≥12 months.

**Figure 2:**
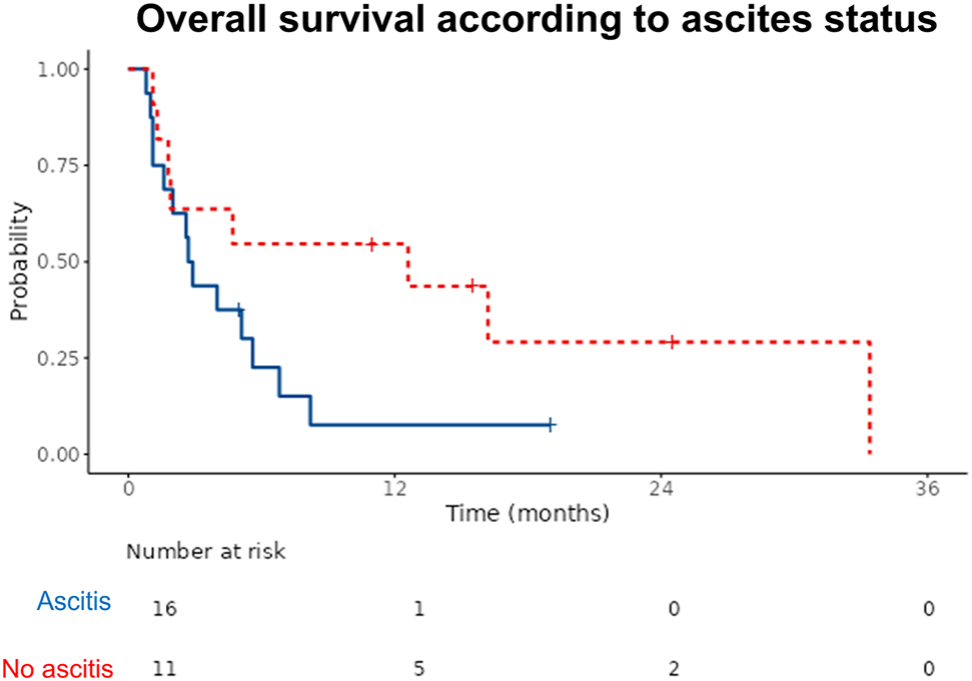
Overall survival according to ascites status. Survival according to presence of ascites. With presence of ascites at the time of surgery (blue line): Median OS 2 months (IQR=6.9), compared to 2 months (IQR=10.8) without (red line), p=0.58.

Postoperative resumption of systemic chemotherapy was associated with improved survival: median OS was 5.6 months (IQR=12.7) for those resuming treatment vs. 1.9 months (IQR=1) for those who did not (p=0.02). Ascites was more frequent in patients with shorter survival (71 vs. 40 %), although the difference did not reach statistical significance (p=0.12) (Figure 2).

## Discussion

This study suggests that surgical intervention for malignant bowel obstruction (MBO) due to advanced peritoneal metastases (PM) may provide clinical benefit in a selected subset of patients. While the overall prognosis remains poor, approximately one-third of our cohort achieved a survival of six months or longer, with 26 % surviving beyond one year. These findings support the notion that palliative surgery can be a viable option when carefully tailored to patient-specific factors.

The role and benefit of surgical therapy in the management of PM-related MBO is debated. The present study displayed a median OS of 4 months overall, with 2 months in patients surviving <6 months, but 14 months in patients surviving >6 months. These figures are similar to those reported by De Boer et al. [8], with a median OS of 119 days, ranging between 48 and 420 days, mirroring the present results. However, De Boer et al. [8] claimed that best supportive care may be a better option than surgery, which is open to debate in light of the results of our presented study. The reason of discrepancy in interpretation may be their higher post-operative complication rate with 8.8 % in-hospital mortality, higher than reported in the present study. A systematic review of the literature reported a median survival of 26–273 days (1–9 months), with high morbidity (6–44 %) and high mortality (6–32 %) [3]. Older data from a single-centre study reported only a third of patients surviving beyond 90 days [9]. Although the current study reported few patients, major morbidity rate (26 %) without in-hopsital mortality, rather short LOS (12 days) may be considered acceptable and in favour of explorative surgery. Optimization of perioperative care within a longstanding enhanced recovery after surgery (ERAS) protocols may also be part of the results [10].

Furthermore, overall prognosis of PM-related MBO without surgical treatment is poor: naso-gastric drainage alone has survival rate of 30–37 days [3], 11]. The relatively low rate of ostomies (19 %) observed in our series reflects a tailored palliative surgical strategy, favoring internal bypass or limited resection whenever feasible, in order to preserve bowel continuity and minimize stoma-related morbidity. For patients in whom surgery was deemed contraindicated, non-operative management including percutaneous endoscopic gastrostomy (PEG) placement was considered to alleviate symptoms and prevent aspiration. These patients were not included in the present cohort.

Our results align with previous literature indicating that selected patients with favorable prognostic indicators – such as limited ascites, preserved functional status, and potential for further systemic treatment – may derive meaningful survival benefit from surgery [7]. Our cohort included a small subgroup of patients (19 %) previously treated with intraperitoneal chemotherapy (PIPAC or HIPEC). While the number is limited, this finding underlines the complexity of therapeutic trajectories in patients with recurrent peritoneal metastases presenting with malignant bowel obstruction. Notably, the ability to resume systemic chemotherapy postoperatively was significantly associated with improved survival. This suggests that surgery should not be considered in isolation but rather as part of a multimodal therapeutic strategy.

Presence of ascites was different between both groups in the present study but the limited number of patients did not allow to reach statistical significance due to type II error. Furthermore, adequate nutritional status, good performance status and life expectancy are factors to consider before surgery [7]. Recently, a scoring system was described based on anatomical considerations (level of obstruction and level of bowel distension) as a help to surgical indications in MBO [12]. While these aspects could be helpful in guiding surgeons and patients to proceed with surgery, patients’ postoperative quality of life data would, however, also be a valuable discriminating factor in choosing an operating approach. This aspect could not be explored in retrospect in our study. We believe that a broad clinical assessment with multi-dimensional assessment, and multidisciplinary counselling is required prior a decision for surgery.

Importantly, our data also highlight that surgical morbidity, while high (33 % overall, 26 % major), did not translate into postoperative mortality. Most patients were able to be discharged from the hospital, with more than half returning home. These results suggest that, when successful, surgery can meaningfully alter the clinical trajectory for selected patients.

It is noteworthy that traditional prognostic markers, including PCI and nutritional status, did not correlate with long-term survival in our cohort. This underscores the limitations of current selection tools and the importance of ongoing clinical judgment and multidisciplinary discussion. The high rate of re-obstruction (52 %) further emphasizes the palliative nature of these procedures and the necessity for clear communication with patients regarding goals of care.

Alternatives to surgery may be endoscopic stenting. While initial success is high [13], long term patency and risks of migration and perforation (especially under systemic chemotherapy) limits its use in MBO. Pre-requisite for endoscopic treatment, is a clearly identified single site, accessible by upper or lower endoscopy [4]. Incidence of return to systemic chemotherapy after surgery was rarely reported in the literature. De Boer et al. reported only 18 % post-operative chemotherapy, compared to 70 % in the present study [3]. While also not reported in systematic reviews [3], 11], 14], high rate of post-operative chemotherapy might contribute on the longer survival described in a subgroup of the present study.

The limitations of the present study are its retrospective nature, the relatively small sample size and heterogeneous underlying primary malignancy. This limited us to observe differences in the subgroups. Moreover, patients’ functional status could not be assessed a posteriori, as well as precise symptoms or quality of life improvements.

Future studies in the management of PM-related MBO should focus on patient-centred outcomes rather than oncological and surgical outcomes, in order to describe precisely the real patients’ end of life journey. Improvement of the medical management and multidisciplinary aspects of patients’ care need also to be further investigated [7]. A multicenter, registry-based, collaborative data collection could represent a future study model in order to further stratify patients groups.

In conclusion, surgery for MBO secondary to PM is associated with substantial perioperative morbidity but may offer symptom relief and meaningful survival, particularly when integrated into a multimodal treatment approach. The ability to resume systemic chemotherapy appears to be a key factor associated with improved outcomes. While careful patient selection remains essential, our findings support the role of palliative surgery as a potentially valuable intervention in this challenging clinical scenario.
